# A randomized, phase 2 study of deoxyuridine triphosphatase inhibitor, TAS-114, in combination with S-1 versus S-1 alone in patients with advanced non-small-cell lung cancer

**DOI:** 10.1007/s10637-020-00930-5

**Published:** 2020-04-03

**Authors:** Nobuyuki Yamamoto, Hidetoshi Hayashi, David Planchard, Teresa Morán, Vanesa Gregorc, Jonathan Dowell, Hiroshi Sakai, Kiyotaka Yoh, Makoto Nishio, Alexis B. Cortot, Karim A. Benhadji, Nital Soni, Jinhong Huang, Lukas Makris, Susana Cedres

**Affiliations:** 1 Third Department of Internal Medicine, Wakayama Medical University, 811-1 Kimiidera, Wakayama, Wakayama Prefecture 641-8509 Japan; 2 Department of Medical Oncology, Kindai University Faculty of Medicine, 377-2 Ohno-Higashi, Osaka-Sayama, Osaka 589-8511 Japan; 3 Department of Medical Oncology, Thoracic Group, Institut Gustave Roussy, 114 rue Édouard- Vaillant, Villejuif Cedex, 94805 France; 4 Medical Oncology, Catalan Institute of Oncology, Hospital Germans Trias i Pujol, Universitat Autonoma de Barcelona (UAB), B-ARGO, Carretera de Canyet s/n, Badalona, Barcelona 08916 Spain; 5 Department of Oncology, Division of Experimental Medicine, IRCCS Ospedale San Raffaele, Via Olgettina, 60, Milano, 20132 Italy; 6 Department of Internal Medicine, University of Texas Southwestern Medical Center, 5323 Harry Hines Blvd, Dallas, TX 75390 USA; 7 Department of Thoracic Oncology, Saitama Cancer Center, 780 Komuro, Ina, Kita-Adachi, Saitama, 362-0806 Japan; 8 Department of Thoracic Oncology, National Cancer Center Hospital East, 6-5-1 Kashiwanoha, Kashiwa, Chiba 277-8577 Japan; 9 Department of Thoracic Medical Oncology, The Cancer Institute Hospital of the Japanese Foundation for Cancer Research, 3-8-31, Ariake, Koto, Tokyo 135-8550 Japan; 10 Thoracic Oncology Department, Centre Hospitalier Universitaire de Lille, 2 Avenue Oscar Lambret, Lille, 59000 France; 11 Department of Clinical Development, Taiho Oncology, Inc, 101 Carnegie Center, Suite 101, Princeton, NJ 08540 USA; 12 Department of Pharmacovigilance, Taiho Pharmaceutical Co., Ltd, 1-27 Kandanishiki-cho, Chiyoda-ku, Tokyo, 101-8444 Japan; 13 Stathmi, Inc, 125 Brownsburg Rd, New Hope, PA 18938 USA; 14 Medical Oncology Department, Vall d´Hebron University Hospital/Vall d´Hebron Institute of Oncology, Passeig de la Vall d’Hebron 119-129, Barcelona, 08035 Spain

**Keywords:** dUTPase, Non-small-cell lung cancer, 5-fluorouracil, Progression-free survival

## Abstract

*Introduction* TAS-114 is a potent inhibitor of deoxyuridine triphosphatase, which is a gatekeeper protein preventing uracil and 5-fluorouracil (5-FU) misincorporation into DNA. TAS-114 has been suggested to enhance the antitumor activity of 5-FU. This randomized, phase 2 study investigated TAS-114 plus S-1 (TAS-114/S-1) vs. S-1 in non-small-cell lung cancer (NSCLC) patients. *Methods* Patients with advanced NSCLC, previously treated with ≥ 2 regimens, were randomized 1:1 to receive TAS-114 (400 mg)/S-1 (30 mg/m^2^) or S-1 (30 mg/m^2^). Progression-free survival (PFS, independent central review) was the primary endpoint. Secondary endpoints included disease control rate (DCR), overall survival (OS), overall response rate (ORR), and safety. *Results* In total, 127 patients received treatment. Median PFS was 3.65 and 4.17 months in the TAS-114/S-1 and S-1 groups, respectively (hazard ratio [HR] 1.16, 95% confidence interval [CI] 0.71–1.88; *P* = 0.2744). DCR was similar between groups (TAS-114/S-1 80.3%, S-1 75.9%) and median OS was 7.92 and 9.82 months for the TAS-114/S-1 and S-1 groups, respectively (HR 1.31, 95% CI 0.80–2.14; *P* = 0.1431). The ORR was higher in the TAS-114/S-1 group than the S-1 group (19.7% vs. 10.3%), and more patients with tumor shrinkage were observed in the TAS-114/S-1 group. Incidence rates of anemia, skin toxicities, and Grade ≥ 3 treatment-related adverse events were higher in the TAS-114/S-1 group compared with the monotherapy group. *Conclusions* Although the TAS-114/S-1 combination improved the response rate, this did not translate into improvements in PFS. *Clinical Trial Registration No.* NCT02855125 (ClinicalTrials.gov) registered on 4 August 2016.

**Electronic supplementary material:**

The online version of this article (10.1007/s10637-020-00930-5) contains supplementary material, which is available to authorized users.

## Introduction

Non-small-cell lung cancer (NSCLC) is the leading cause of cancer incidence and cancer-related mortality worldwide [1]. Recently, the development of immunotherapy and its combination with cytotoxic chemotherapies has garnered new options for therapeutics in advanced NSCLC [2]. However, despite recent advances, most patients still progress after treatment, and cytotoxic chemotherapies remain essential in NSCLC treatment algorithms [3, 4]. Thus, highly efficacious, well-tolerated chemotherapies are still warranted.

S-1 is an oral 5-fluorouracil (5-FU) derivative that is widely used for the treatment of various solid tumors, including NSCLC [5]. Recently, a phase 3 study of S-1 monotherapy demonstrated non-inferiority compared with docetaxel in patients with NSCLC; thus, S-1 is considered a standard chemotherapy option in East Asia [6].

TAS-114 is a potent inhibitor of deoxyuridine triphosphatase (dUTPase), which is a gatekeeper protein that prevents uracil and 5-FU misincorporation into DNA. TAS-114 inhibits the conversion of deoxyuridine triphosphate (dUTP) and 5-fluoro-dUTP (FdUTP) into their monophosphate forms. TAS-114 itself does not have antitumor activity; however, when it is combined with 5-FU derivatives, it can increase the amount of FdUTP in the tumor for selective incorporation into DNA, thus enhancing the antitumor effect [7, 8].

Two previous phase 1 studies of TAS-114 combined with S-1, conducted separately in Europe and Japan, concluded that the recommended dose was TAS-114 240 mg/m^2^ plus S-1 30 mg/m^2^. The combination of TAS-114 and S-1 demonstrated promising antitumor activity, with a manageable safety profile in heavily pretreated solid tumors. The overall response rate (ORR), including unconfirmed partial response (PR), in NSCLC was approximately 30% [9, 10]. Based on these results, we conducted a randomized phase 2 study to investigate the combination of TAS-114 with S-1 vs. S-1 monotherapy in patients with advanced NSCLC.

## Materials and methods

### Study design

This was an open-label, multicenter, international, randomized phase 2 study conducted at 28 sites across five countries (Japan, France, Spain, Italy, and the United States). Eligible patients were enrolled by the investigators and randomly assigned to TAS-114 plus S-1 (TAS-114/S-1) or S-1 alone in a 1:1 ratio. The random allocation sequence was generated, and treatment assignment performed centrally using a dynamic allocation method (biased coin) via an interactive voice/web response system. Patients were stratified by histological subtype (squamous vs. non-squamous) and region (Europe/United States vs. Japan).

The study was approved by independent ethics committees or institutional review boards at each site. The study conduct was in accordance with Good Clinical Practice Guidelines and the Declaration of Helsinki. All patients provided written informed consent before enrollment (ClinicalTrials.gov identifier NCT02855125).

### Patients

Key eligibility criteria were age ≥ 18 years (≥ 20 years in Japan), histologically confirmed advanced or metastatic NSCLC; prior treatment with at least two systemic therapies (one of which must be platinum doublet and either pemetrexed, docetaxel, or immunotherapy) for advanced or metastatic NSCLC; Eastern Cooperative Oncology Group performance status (ECOG PS) of 0–1; adequate bone marrow function (absolute neutrophil count ≥ 1500/mm^3^, hemoglobin ≥ 10.0 g/dL, platelets ≥ 100,000/mm^3^); adequate liver function (total bilirubin ≤ 1.5 × upper limit of normal [ULN], aspartate aminotransferase/alanine aminotransferase ≤ 3 × ULN [in patients with existent liver metastases, ≤ 5 × ULN]); and adequate renal function (calculated creatinine clearance ≥ 50 mL/min [Cockcroft-Gault]). All eligibility criteria are listed in Online Resource Text S1.

### Treatment

TAS-114 400 mg and/or S-1 30 mg/m^2^ were concurrently administered orally twice daily under fasting conditions in a schedule consisting of 14 consecutive days of treatment followed by a 7-day rest. TAS-114 dosing was switched from body surface area (BSA)-based to a flat-fixed dose to avoid the risk of dosing errors. TAS-114 400 mg was equivalent to 240 mg/m^2^, which is the recommended dose of TAS-114 for an overall mean BSA of 1.69 m^2^ based on the results of the two phase 1 studies [9, 10]. In these studies, there was no correlation between the oral clearance of TAS-114 and BSA. Treatment was repeated every 3 weeks until disease progression, intolerable toxicities, or withdrawal of consent. No crossover was permitted.

### Endpoints and study assessments

The primary endpoint was progression-free survival (PFS) as determined by independent central review (ICR). Secondary endpoints were ORR, disease control rate (DCR), overall survival (OS), and safety. PFS was defined as the time from randomization to the date of first radiologic disease progression or death, whichever occurred first. ORR was defined as the proportion of patients with complete response (CR) or PR. DCR was defined as the proportion of patients with CR, PR, or stable disease. OS was defined as the time from randomization to death. Tumor shrinkage for each patient and its correlation with PFS by ICR was assessed as an exploratory analysis.

Tumor assessments were performed every 6 weeks from the first study drug administration until disease progression according to the RECIST version 1.1. Assessment results were evaluated by independent central radiologists (Medpace Imaging Core Lab, Ohio, USA) and by the investigators at each site.

Adverse events (AEs) were reported using the verbatim term, and the severity was graded based on the National Cancer Institute Common Terminology Criteria for Adverse Events version 4.03. AEs were coded by preferred term according to the Medical Dictionary for Regulatory Activities (MedDRA^®^: trademark is registered by the International Federation of Pharmaceutical Manufacturers & Associations on behalf of the International Council for Harmonisation) version 19.0.

### Statistical analysis

The sample size was estimated based on PFS. A total sample size of 124 patients was required to provide 60 events (progressive disease or death) by ICR for the primary analysis, which corresponds to 71 events based on investigator review under the assumption that the discrepancy in progressive disease events between ICR and investigator review is 15%. Based on the results of previous clinical trials [9–13], the median PFS in the S-1 group was estimated to be 2.2 months, and 4.2 months in the TAS-114/S-1 group, corresponding to a hazard ratio (HR) of 0.524, with 80% statistical power and a one-sided significance level of 0.05.

The PFS and OS analyses were conducted on an intent-to-treat (ITT) population, of all patients randomized in the study, regardless of whether they received any study treatment or not. The ORR was analyzed based on the tumor response-evaluable population, defined as all patients in the ITT population with measurable disease (at least one target lesion) at baseline and with at least one tumor evaluation. We performed a sensitivity analysis in which PFS, ORR, and DCR were also analyzed based on the investigator’s assessment. The safety analysis was conducted on an as-treated population, defined as all patients who received at least one dose of the study drug.

PFS and OS were estimated using the Kaplan–Meier method; HRs were estimated using a Cox proportional hazards model stratified by geographical region and histological subtypes. Medians (with 95% confidence intervals [CIs]) were calculated using the Brookmeyer-Crowley method. For between-group comparisons, the stratified log-rank test with a one-sided significance level of 5% was used for PFS and OS, and Fisher’s exact test for ORR and DCR. All statistical analyses were performed using SAS software version 9.4 in the UNIX environment (SAS Institute Inc., North Carolina, USA).

## Results

### Patients

**Fig. 1:**
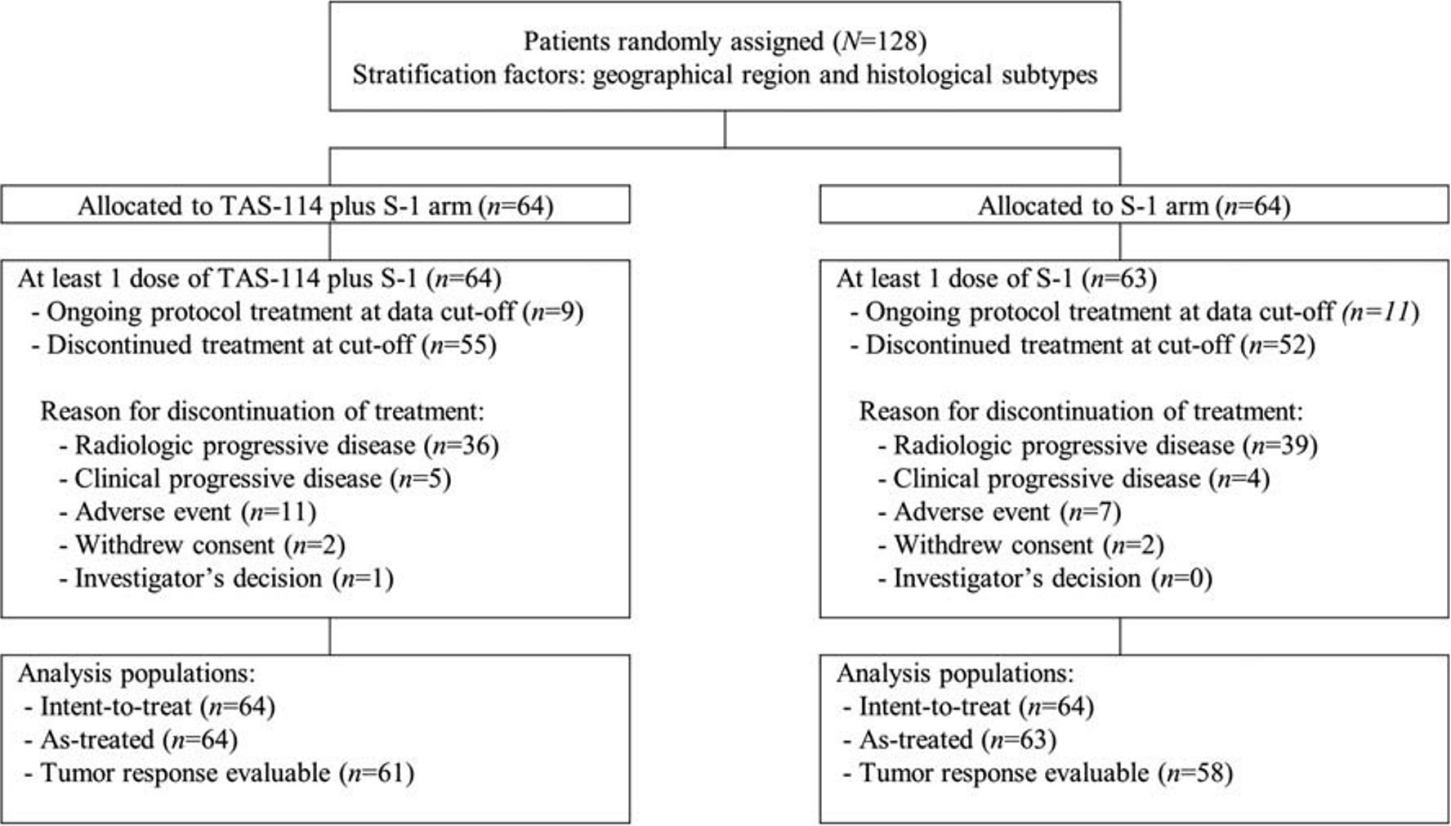
CONSORT flow diagram

From August 2016 to July 2017, a total of 128 patients were randomly assigned to treatment: 64 patients each were assigned to the TAS-114/S-1 and S-1 groups (Fig. 1). A total of 127 patients received at least one study treatment; one patient in the S-1 group was found ineligible, and thus, discontinued before the initial study drug administration. Twenty patients were receiving treatment by the cut-off date for primary analysis (30 September 2017). Baseline characteristics were generally balanced in the two groups, except for sex, ECOG PS, and epidermal growth factor receptor (*EGFR*) mutation status (Table 1). More than half of the patients had received a prior systemic regimen as fourth-line or further therapy. Over 70% of patients had received prior programmed death 1/programmed death-ligand 1 antibodies.

**Table 1:** Baseline demographic characteristics (intent-to-treat population)

Characteristic	TAS-114/S-1 (*n* = 64)	S-1 (*n* = 64)
Age (years), median	65.5	64.0
Age group, *n* (%)		
< 65 years	28 (43.8)	33 (51.6)
≥ 65 years	36 (56.3)	31 (48.4)
Sex, *n* (%)		
Male	41 (64.1)	48 (75.0)
Female	23 (35.9)	16 (25.0)
Region, *n* (%)		
Western	34 (53.1)	34 (53.1)
Asian	30 (46.9)	30 (46.9)
ECOG PS, *n* (%)		
0	12 (18.8)	17 (26.6)
1	52 (81.3)	47 (73.4)
Histology subtype, *n* (%)		
Squamous cell carcinoma	14 (21.9)	14 (21.9)
Adenocarcinoma	48 (75.0)	48 (75.0)
Large cell carcinoma	0	1 (1.6)
Carcinoid tumor	1 (1.6)	0
Other	1 (1.6)	1 (1.6)
*EGFR* mutation status, *n* (%)		
Positive	7 (10.9)	12 (18.8)
Negative	46 (71.9)	38 (59.4)
Unknown	11 (17.2)	14 (21.9)
ALK translocation status, *n* (%)		
Positive	2 (3.1)	0
Negative	44 (68.8)	43 (67.2)
Unknown	18 (28.1)	21 (32.8)
Brain metastases, *n* (%)	6 (9.4)	7 (11.0)
Number of prior systemic regimens, *n* (%)		
1	0	0
2	24 (37.5)	19 (29.7)
3	19 (29.7)	17 (26.6)
4	14 (21.9)	15 (23.4)
≥ 5	7 (10.9)	13 (20.3)
Main prior systemic anti-cancer agent, *n* (%)		
Platinum	64 (100)	64 (100)
PD-1/PD-L1 antibodies	46 (71.9)	49 (76.6)
Pemetrexed	48 (75.0)	47 (73.4)
Docetaxel	33 (51.6)	38 (59.4)
EGFR-TKI	7 (11.0)	11 (17.2)
ALK-TKI	2 (3.1)	0

ALK, anaplastic lymphoma kinase; ECOG PS, Eastern Cooperative Oncology Group performance status; EGFR, epidermal growth factor receptor; PD-1, programmed death 1; PD-L1, programmed death ligand 1; TKI, tyrosine kinase inhibitor

### Treatment

In the TAS-114/S-1 and S-1 groups, patients received medians of 4.0 (range, 1–14) and 3.0 (range, 1–12) treatment cycles, respectively. In the TAS-114/S-1 group, the medians for relative dose intensity (RDI) of TAS-114 and S-1 were 85.9% and 84.8%, respectively. In the S-1 group, the median RDI was 95.5%. By the data cut-off date, 107 patients had discontinued study treatment. In both groups, the main reason for discontinuation was disease progression. After study discontinuation, 54.5% and 65.4% of patients in the TAS-114/S-1 and S-1 groups, respectively, received other anticancer treatments. The median duration for survival follow-up was 9.03 months.

### Efficacy

**Fig. 2:**
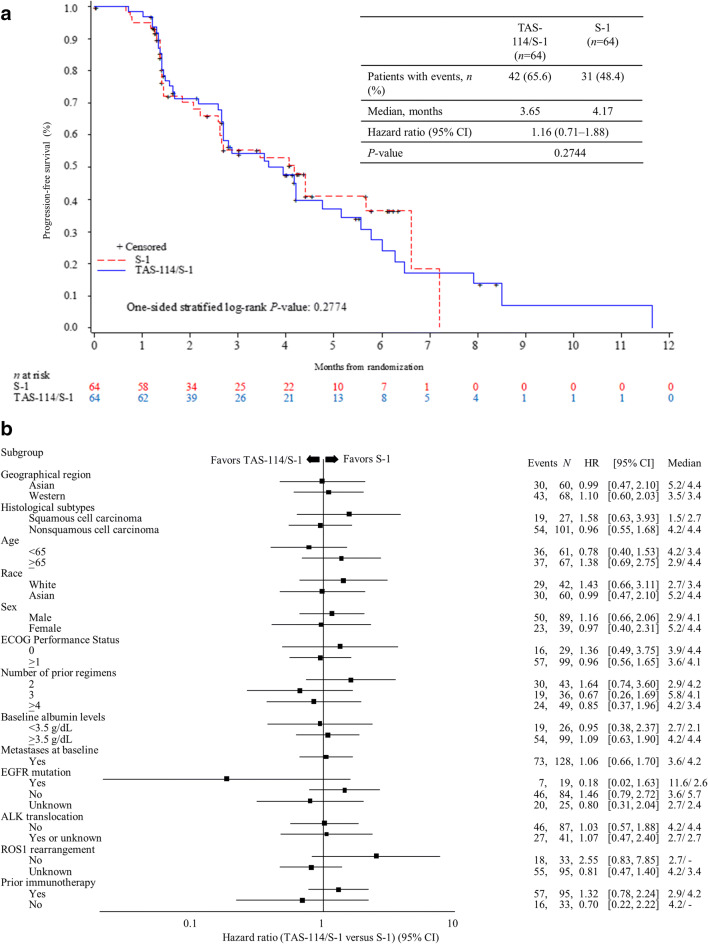
**(a)** Progression-free survival by ICR (intent-to-treat population), **(b)** forest plot of hazard ratios for treatment effect on progression-free survival (ICR). ALK, anaplastic lymphoma kinase; CI, confidence interval; ECOG, Eastern Cooperative Oncology Group; EGFR, epidermal growth factor receptor; HR, hazard ratio; ICR, independent central review; ROS1, ROS proto-oncogene 1, receptor tyrosine kinase

**Fig. 3:**
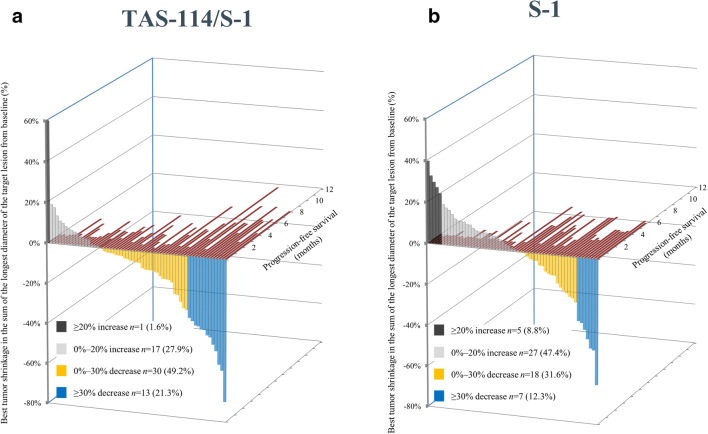
Individual tumor shrinkage and progression-free survival (independent central review) in the **(a)** TAS-114/S-1 group and **(b)** S-1 group

At the data cut-off date, 73 PFS events were observed by ICR. The median PFS was 3.65 months in the TAS-114/S-1 group and 4.17 months in the S-1 group (HR 1.16, 95% CI 0.71–1.88; *P* = 0.2744) (Fig. 2a). In the subgroup analyses, the data were generally similar to that of the overall population (Fig. 2b). The DCR was similar between TAS-114/S-1 and S-1 groups (80.3% vs. 75.9%), whereas patients in the TAS-114/S-1 group showed higher ORR than those in the S-1 group (19.7% vs. 10.3%) (Table 2). Additionally, Fig. 3a and b shows the relationship between tumor shrinkage and PFS by RECIST for each patient. Investigator-assessed results were 3.48 months vs. 2.66 months for PFS (HR 0.79, 95% CI 0.52–1.21; *P* = 0.1352), 78.7% vs. 59.3% for DCR, and 19.7% vs. 10.2% for ORR (Table 2; Online Resource Figures S1 and S2).

**Table 2:** Overall response rate and disease control rate by ICR and INV (tumor response evaluable population)

	ICR		INV	
	TAS-114/S-1 (*n* = 61)	S-1 (*n* = 58)	TAS-114/S-1 (*n* = 61)	S-1 (*n* = 59)
Overall response rate, % (95% CI)	19.7 (10.6–31.8)	10.3 (4.0–21.5)	19.7 (10.6–31.8)	10.2 (3.9–21.2)
CR	1 (1.6)	0	0	0
PR	11 (18.0)	6 (10.3)	12 (19.7)	6 (10.2)
SD	37 (60.7)	38 (65.5)	36 (59.0)	29 (49.2)
PD	12 (19.7)	13 (22.4)	13 (21.3)	23 (39.0)
Disease control rate (CR + PR + SD), % (95% CI)	80.3 (68.2–89.4)	75.9 (64.2–87.3)	78.7 (66.3–88.1)	59.3 (46.6–73.0)

CI, confidence interval; CR, complete response; ICR, independent central review; INV, Investigator review; PR, partial response; PD, progressive disease; SD, stable disease

The data cut-off for OS was 30 November 2017, at which time 65 events were observed. The median OS was 7.92 in the TAS-114/S-1 group and 9.82 months in the S-1 group (HR 1.31, 95% CI 0.80–2.14; *P* = 0.1431) (Online Resource Figures S3 and S4).

### Safety

Treatment-related AEs (TRAEs) occurring in ≥ 10% of patients are shown in Table 3. The incidence rates of anemia, skin toxicities, and Grade ≥ 3 TRAEs were higher in the TAS-114/S-1 group compared with the monotherapy group. Serious TRAEs were reported in 17.2% of patients in the TAS-114/S-1 group, and in 6.7% in the S-1 group. In the TAS-114/S-1 group, two patients each reported treatment-related serious anemia (3.1%), diarrhea (3.1%), and maculo-papular rash (3.1%) of any grade. More patients in the TAS-114/S-1 group had dose reductions/interruptions (mainly due to skin toxicities) than patients in the S-1 group (reductions: 31.3% vs. 11.1%; interruptions: 45.3% vs. 14.3%) (Online Resource Table S1). No treatment-related death was reported.

**Table 3:** Treatment-related adverse events (as-treated population)

	TAS-114/S-1 (*n* = 64)		S-1 (*n* = 63)	
	All Grades	≥Grade 3	All Grades	≥Grade 3
Event	*n* (%)	*n* (%)	*n* (%)	*n* (%)
All	60 (93.8)	35 (54.7)	57 (90.5)	10 (15.9)
Hematology				
Anemia	37 (57.8)	14 (21.9)	10 (15.9)	0
White blood cell count decreased	10 (15.6)	2 (3.1)	5 (7.9)	0
Platelet count decreased	8 (12.5)	1 (1.6)	6 (9.5)	0
Neutrophil count decreased	6 (9.4)	2 (3.1)	7 (11.1)	1 (1.6)
Non-hematology				
Decreased appetite	25 (39.1)	3 (4.7)	22 (34.9)	1 (1.6)
Nausea	20 (31.3)	0	20 (31.7)	1 (1.6)
Diarrhea	18 (28.1)	2 (3.1)	12 (19.0)	1 (1.6)
Skin hyperpigmentation	18 (28.1)	0	11 (17.5)	0
Maculo-papular rash	18 (28.1)	4 (6.3)	2 (3.2)	0
Asthenia	17 (26.6)	4 (6.3)	9 (14.3)	2 (3.2)
Rash	15 (23.4)	5 (7.8)	3 (4.8)	0
Vomiting	10 (15.6)	0	11 (17.5)	2 (3.2)
Pruritus	10 (15.6)	0	7 (11.1)	0
Stomatitis	9 (14.1)	1 (1.6)	4 (6.3)	0
Malaise	8 (12.5)	0	5 (7.9)	0
Fatigue	6 (9.4)	1 (1.6)	9 (14.3)	1 (1.6)
Dry skin	9 (14.1)	0	5 (7.9)	0

## Discussion

The present randomized study is the first to evaluate the effect of a dUTPase inhibitor in combination with a 5-FU derivative. Favorable tumor response and shrinkage trends were observed in the TAS-114/S-1 group; however, they did not translate into an improved PFS. No difference in duration of response was observed, and the early progressive disease (< 3 months) rate was similar across groups. A possible reason is that more patients in the TAS-114/S-1 group experienced dose reductions/interruptions due to AEs, and these results may have affected the primary endpoint result.

According to the investigator review, the median PFS was 3.48 months for the TAS-114/S-1 group and 2.66 months for the S-1 group (95 PFS events). In the S-1 group, a significant difference in the median PFS (1.51-month difference) was observed between the ICR and investigator review, in contrast with the TAS-114/S-1 group (0.17-month difference). This may be a result of treatment bias owing to lack of blinding. In fact, the number of censored cases was higher in the S-1 group by ICR; therefore, the PFS in the S-1 group was presumably influenced by informative censoring [14].

The OS result was immature at the data cut-off, but was lower in the TAS-114/S-1 group (not statistically significant). This difference between groups might have resulted from the percentage and timing of patients receiving anticancer treatment after discontinuation, and some imbalances in patient background characteristics that are known to be prognostic factors, such as *EGFR* mutation [15, 16]. Based on the efficacy and safety results, the sponsor decided to terminate the TAS-114/S-1 combination, and all five patients remaining in TAS-114 treatment were switched to S-1 alone after the primary analysis.

Although the patient numbers were very limited (7 vs. 12 patients), the subgroup analysis showed that *EGFR*-mutant patients tended to achieve longer PFS (11.6 months vs. 2.6 months; HR 0.18; 95% CI 0.02–1.63) and OS (not reached vs. 10.6 months; HR 0.12; 95% CI 0.01–0.99) in the TAS-114/S-1 group vs. the S-1 group. It has been reported that advanced NSCLC *EGFR*-mutant patients present defective DNA-repair functions [17, 18]. In a preclinical model, suppression of DNA repair proteins increased its sensitivity to TAS-114 combined with the 5-FU metabolite 5-fluoro-2’-deoxyuridine [19]. Thus, it is possible that the present favorable effect in patients with *EGFR* mutations in the TAS-114/S-1 group resulted from increased uracil and 5-FU incorporation into DNA.

The overall safety profile in the TAS-114/S-1 group was consistent with previous phase 1 studies; anemia and skin toxicities were more commonly observed in this group [9, 10]. These toxicities were considered manageable by dose modification and standard symptomatic treatment and were presumed to result from the combination of TAS-114 and S-1. In exploratory analysis, patients with anemia and/or skin toxicities in the TAS-114/S-1 group showed a tendency toward longer PFS compared with patients without these toxicities (Online Resource Text S2). Moreover, anemia was also commonly reported with DNA-damaging agents; it is possible that the frequent anemia reported with TAS-114/S-1 was attributed to increased DNA damage [20, 21].

The present study had several limitations. These include the relatively small sample size that may have prevented the identification of some group differences, the open-label design that may have introduced bias from the investigators, and the background characteristic imbalances that may have affected the primary endpoint results.

## Conclusions

Although the TAS-114/S-1 combination did result in a higher response rate and tumor shrinkage in this advanced NSCLC population, it did not improve PFS or OS.

## Electronic supplementary material

ESM 1 (PDF 2213 kb)

## Data Availability

Anonymized, patient-level, analyzable datasets will not be shared according to the Sponsor policy on data sharing (https://www.taiho.co.jp/en/science/policy/clinical_trial_information_disclosure_policy/index.html).
