# Supplementary material for: A randomized, phase 2 study of deoxyuridine triphosphatase inhibitor, TAS-114, in combination with S-1 versus S-1 alone in patients with advanced non-small-cell lung cancer
Source: Invest New Drugs. 2020 Apr 3;38(5):1588–97. doi: 10.1007/s10637-020-00930-5 (PMC7497678; doi:10.1007/s10637-020-00930-5)
Supplement: Supplementary file 1 — (PDF 2213 kb) [file 10637_2020_930_MOESM1_ESM.pdf]

Supplementary Figure S1. Progression-free survival as assessed by the investigators (intent-to-treat population)
CI, confidence interval

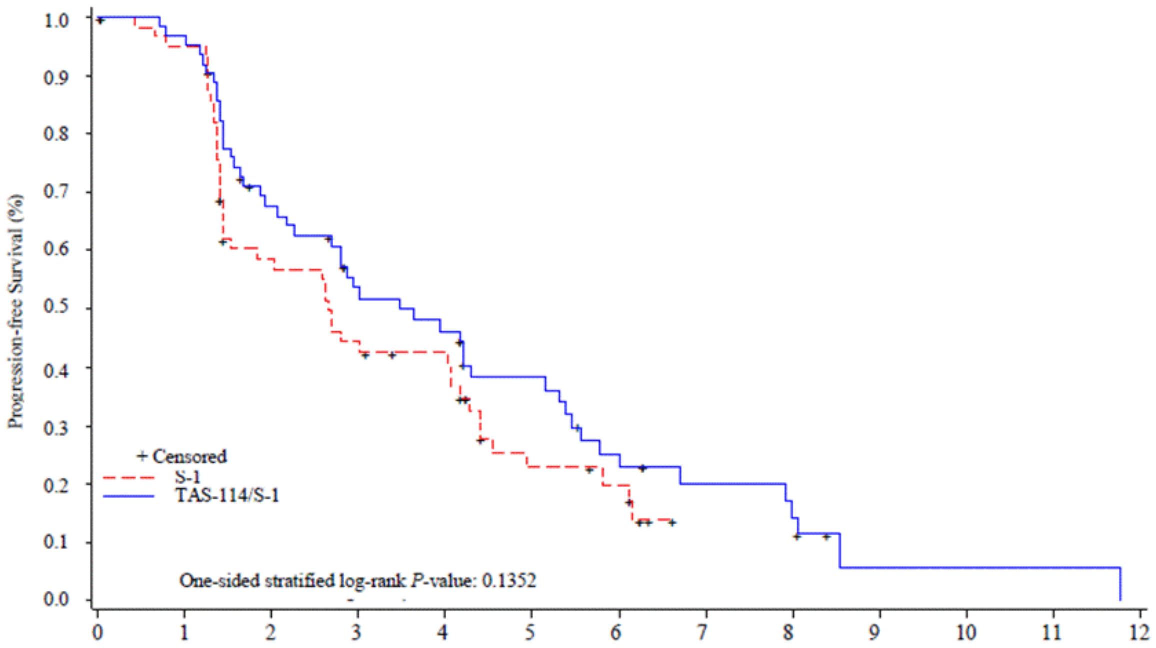

N at Risk													
S-1	64	58	33	25	22	9	7	0	0	0	0	0	0
TAS-114/S-1	64	61	40	29	25	18	11	7	5	1	1	1	0

Article title: A randomized, phase 2 study of deoxyuridine triphosphatase inhibitor, TAS-114, in combination with S-1 versus S-1 alone in patients with advanced non-small-cell lung cancer

Journal name: Investigational New Drugs

Author names: Nobuyuki Yamamoto, Hidetoshi Hayashi, David Planchard, Teresa Morán, Vanesa Gregorc, Jonathan Dowell, Hiroshi Sakai, Kiyotaka Yoh, Makoto Nishio, Alexis B. Cortot, Karim A. Benhadji, Nital Soni, Jinhong Huang, Lukas Makris, Susana Cedres

Corresponding author: Dr. Nobuyuki Yamamoto, Third Department of Internal Medicine, Wakayama Medical University Hospital, 811-1 Kimiidera, Wakayama, Wakayama Prefecture 641-8509, Japan

TEL: +81-73-441-0619

E-mail address: nbyamamo@wakayama-med.ac.jp

Supplementary Figure S2. Forest plot of HRs for treatment effect on PFS as assessed by the investigators

ALK, anaplastic lymphoma kinase; CI, confidence interval; ECOG, Eastern Cooperative Oncology Group; EGFR, epidermal growth factor receptor; HR, hazard ratio; PFS, progression-free survival; ROS1, ROS proto-oncogene 1, receptor tyrosine kinase

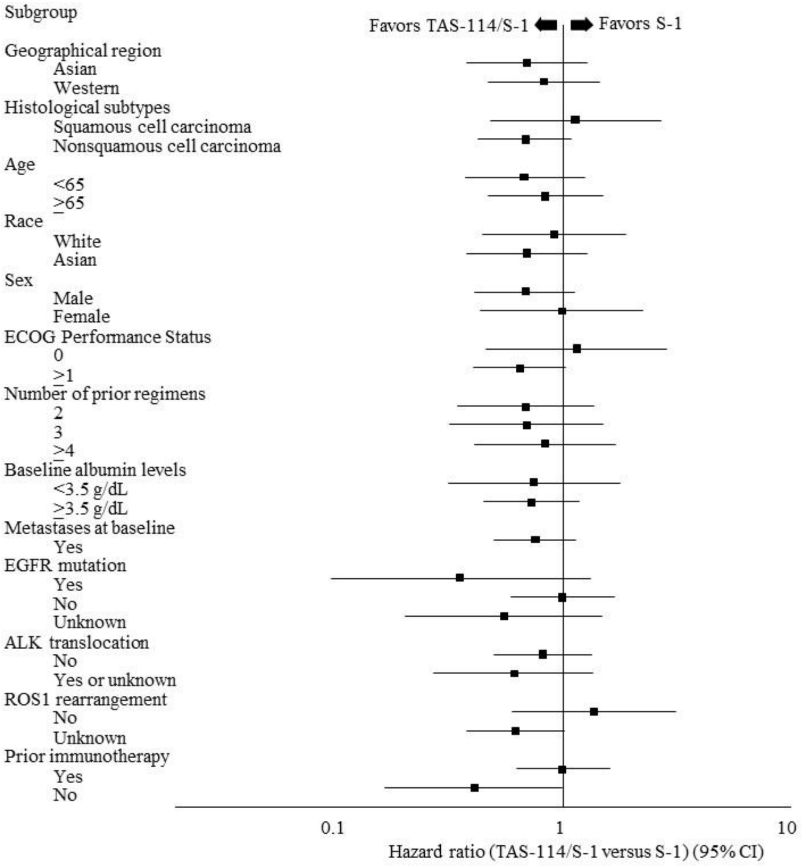

Subgroup	Events	*N*	HR	[95% CI]	Median
Geographical region					
Asian	46,	60,	0.69	[0.38, 1.28]	3.5/ 2.7
Western	49,	68,	0.83	[0.47, 1.47]	3.6/ 2.7
Histological subtypes					
Squamous cell carcinoma	22,	27,	1.14	[0.48, 2.71]	1.9/ 1.4
Nonsquamous cell carcinoma	73,	101,	0.68	[0.42, 1.10]	4.2/ 2.7
Age					
<65	44,	61,	0.68	[0.37, 1.24]	5.3/ 2.6
≥65	51,	67,	0.84	[0.47, 1.50]	3.0/ 2.8
Race					
White	31,	42,	0.91	[0.44, 1.90]	2.7/ 2.7
Asian	46,	60,	0.69	[0.38, 1.28]	3.5/ 2.7
Sex					
Male	67,	89,	0.68	[0.41, 1.13]	3.5/ 2.6
Female	28,	39,	0.99	[0.44, 2.25]	4.2/ 2.9
ECOG Performance Status					
0	19,	29,	1.15	[0.46, 2.86]	2.8/ 4.0
≥1	76,	99,	0.65	[0.40, 1.04]	3.6/ 2.0
Number of prior regimens					
2	35,	43,	0.69	[0.34, 1.39]	2.9/ 2.1
3	28,	36,	0.69	[0.32, 1.50]	4.3/ 3.0
≥4	32,	49,	0.84	[0.41, 1.72]	3.5/ 2.6
Baseline albumin levels					
<3.5 g/dL	22,	26,	0.75	[0.31, 1.80]	1.6/ 1.4
≥3.5 g/dL	72,	99,	0.73	[0.45, 1.18]	3.9/ 2.8
Metastases at baseline					
Yes	95,	128,	0.75	[0.50, 1.14]	3.5/ 2.7
EGFR mutation					
Yes	13,	19,	0.36	[0.10, 1.32]	4.3/ 1.4
No	62,	84,	1.00	[0.59, 1.68]	3.0/ 2.8
Unknown	20,	25,	0.55	[0.20, 1.49]	2.2/ 2.0
ALK translocation					
No	66,	87,	0.82	[0.50, 1.34]	3.5/ 2.6
Yes or unknown	29,	41,	0.61	[0.27, 1.36]	4.2/ 4.0
ROS1 rearrangement					
No	26,	33,	1.37	[0.60, 3.15]	2.8/ 4.1
Unknown	69,	95,	0.62	[0.38, 1.03]	4.2/ 2.6
Prior immunotherapy					
Yes	72,	95,	1.00	[0.62, 1.61]	3.0/ 2.7
No	23,	33,	0.41	[0.17, 0.99]	5.3/ 1.4

Article title: A randomized, phase 2 study of deoxyuridine triphosphatase inhibitor, TAS-114, in combination with S-1 versus S-1 alone in patients with advanced non-small-cell lung cancer

Journal name: Investigational New Drugs

Author names: Nobuyuki Yamamoto, Hidetoshi Hayashi, David Planchard, Teresa Morán, Vanesa Gregorc, Jonathan Dowell, Hiroshi Sakai, Kiyotaka Yoh, Makoto Nishio, Alexis B. Cortot, Karim A. Benhadji, Nital Soni, Jinhong Huang, Lukas Makris, Susana Cedres

Corresponding author: Dr. Nobuyuki Yamamoto, Third Department of Internal Medicine, Wakayama Medical University Hospital, 811-1 Kimiidera, Wakayama, Wakayama Prefecture 641-8509, Japan

TEL: +81-73-441-0619

E-mail address: nbyamamo@wakayama-med.ac.jp

Supplementary Figure S3. Overall survival (intent-to-treat population)

CI, confidence interval

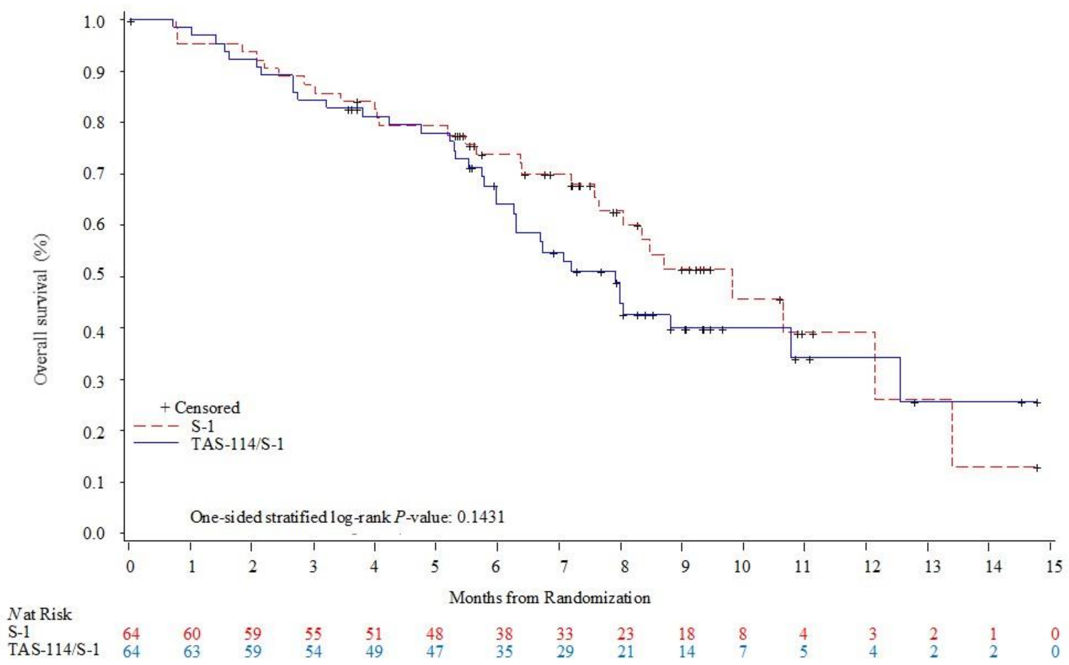

Article title: A randomized, phase 2 study of deoxyuridine triphosphatase inhibitor, TAS-114, in combination with S-1 versus S-1 alone in patients with advanced non-small-cell lung cancer

Journal name: Investigational New Drugs

Author names: Nobuyuki Yamamoto, Hidetoshi Hayashi, David Planchard, Teresa Morán, Vanesa Gregorc, Jonathan Dowell, Hiroshi Sakai, Kiyotaka Yoh, Makoto Nishio, Alexis B. Cortot, Karim A. Benhadji, Nital Soni, Jinhong Huang, Lukas Makris, Susana Cedres

Corresponding author: Dr. Nobuyuki Yamamoto, Third Department of Internal Medicine, Wakayama Medical University Hospital, 811-1 Kimiidera, Wakayama, Wakayama Prefecture 641-8509, Japan

TEL: +81-73-441-0619

E-mail address: nbyamamo@wakayama-med.ac.jp

Supplementary Figure S4. Forest plot of HRs for treatment effect on OS

ALK, anaplastic lymphoma kinase; CI, confidence interval; ECOG, Eastern Cooperative Oncology Group; EGFR, epidermal growth factor receptor; HR, hazard ratio; OS, overall survival; ROS1, ROS proto-oncogene 1, receptor tyrosine kinase

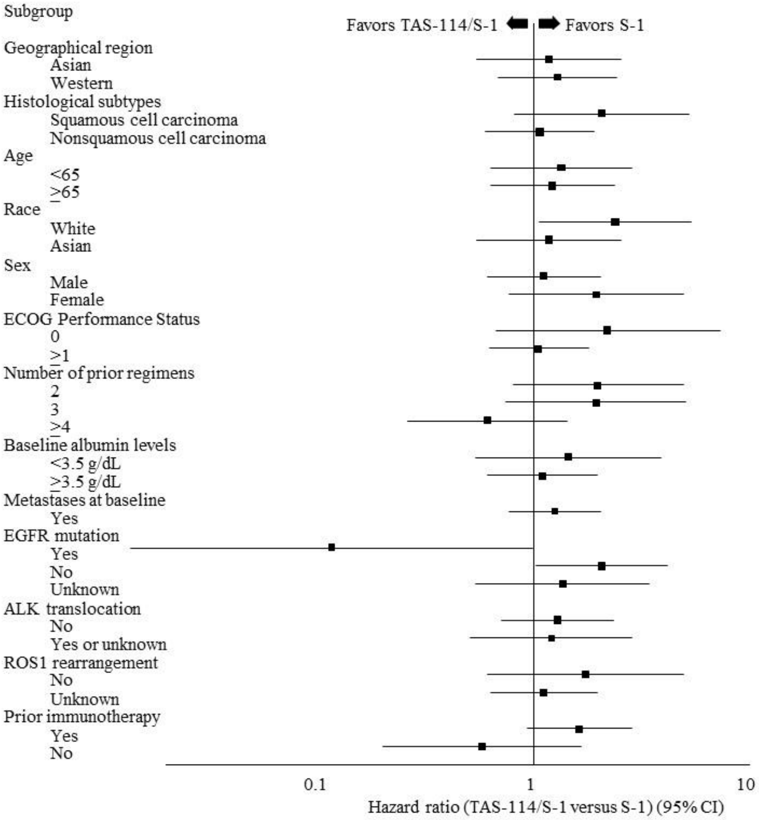

Subgroup	Events	*N*	HR	[95% CI]	Median
Geographical region					
Asian	26,	60,	1.18	[0.55, 2.56]	10.8/10.6
Western	39,	68,	1.30	[0.69, 2.45]	6.7/ 8.5
Histological subtypes					
Squamous cell carcinoma	19,	27,	2.08	[0.82, 5.31]	3.8/ 7.7
Nonsquamous cell carcinoma	46,	101,	1.07	[0.60, 1.92]	10.8/10.6
Age					
<65	28,	61,	1.35	[0.63, 2.87]	10.8/10.6
≥65	37,	67,	1.23	[0.64, 2.38]	7.9/ 8.5
Race					
White	26,	42,	2.41	[1.07, 5.44]	6.0/ 8.7
Asian	26,	60,	1.18	[0.55, 2.56]	10.8/10.6
Sex					
Male	43,	89,	1.12	[0.62, 2.05]	8.0/ 8.7
Female	22,	39,	1.97	[0.77, 5.02]	6.3/10.6
ECOG Performance Status					
0	11,	29,	2.21	[0.67, 7.35]	6.3/10.6
≥1	54,	99,	1.06	[0.62, 1.82]	7.9/ 8.3
Number of prior regimens					
2	21,	43,	2.00	[0.80, 4.97]	7.1/ -
3	20,	36,	1.95	[0.75, 5.11]	7.2/ -
≥4	24,	49,	0.61	[0.26, 1.44]	10.8/ 9.8
Baseline albumin levels					
<3.5 g/dL	18,	26,	1.45	[0.54, 3.91]	6.3/ 6.1
≥3.5 g/dL	46,	99,	1.10	[0.62, 1.97]	8.8/ 9.8
Metastases at baseline					
Yes	65,	128,	1.27	[0.78, 2.07]	7.9/ 9.8
EGFR mutation					
Yes	9,	19,	0.12	[0.01, 0.99]	- /10.6
No	38,	84,	2.08	[1.04, 4.16]	7.9/13.4
Unknown	18,	25,	1.37	[0.54, 3.46]	6.3/ 5.5
ALK translocation					
No	43,	87,	1.29	[0.71, 2.36]	7.9/ 9.8
Yes or unknown	22,	41,	1.21	[0.51, 2.89]	6.7/ 8.3
ROS1 rearrangement					
No	17,	33,	1.76	[0.62, 5.01]	6.3/ 8.5
Unknown	48,	95,	1.12	[0.64, 1.97]	8.0/ 9.8
Prior immunotherapy					
Yes	50,	95,	1.63	[0.93, 2.86]	6.7/ 8.7
No	15,	33,	0.58	[0.20, 1.67]	10.8/10.6

Article title: A randomized, phase 2 study of deoxyuridine triphosphatase inhibitor, TAS-114, in combination with S-1 versus S-1 alone in patients with advanced non-small-cell lung cancer

Journal name: Investigational New Drugs

Author names: Nobuyuki Yamamoto, Hidetoshi Hayashi, David Planchard, Teresa Morán, Vanesa Gregorc, Jonathan Dowell, Hiroshi Sakai, Kiyotaka Yoh, Makoto Nishio, Alexis B. Cortot, Karim A. Benhadji, Nital Soni, Jinhong Huang, Lukas Makris, Susana Cedres

Corresponding author: Dr. Nobuyuki Yamamoto, Third Department of Internal Medicine, Wakayama Medical University Hospital, 811-1 Kimiidera, Wakayama, Wakayama Prefecture 641-8509, Japan

TEL: +81-73-441-0619

E-mail address: nbyamamo@wakayama-med.ac.jp

Supplementary Table S1. The rates of dose reduction and dose interruption

	**TAS-114/S-1 (*n*=64)**	**S-1 (*n*=63)**
Dose reduction	20 (31.3)	7 (11.1)
AE	19 (95.0)	5 (71.4)
Other	1 (5.0)	3 (42.9)
Dose interruption	29 (45.3)	9 (14.3)
AE	27 (93.1)	8 (88.9)
Other	5 (17.2)	2 (22.2)

AE, adverse event

Article title: A randomized, phase 2 study of deoxyuridine triphosphatase inhibitor, TAS-114, in combination with S-1 versus S-1 alone in patients with advanced non-small-cell lung cancer

Journal name: Investigational New Drugs

Author names: Nobuyuki Yamamoto, Hidetoshi Hayashi, David Planchard, Teresa Morán, Vanesa Gregorc, Jonathan Dowell, Hiroshi Sakai, Kiyotaka Yoh, Makoto Nishio, Alexis B. Cortot, Karim A. Benhadji, Nital Soni, Jinhong Huang, Lukas Makris, Susana Cedres

Corresponding author: Dr. Nobuyuki Yamamoto, Third Department of Internal Medicine, Wakayama Medical University Hospital, 811-1 Kimiidera, Wakayama, Wakayama Prefecture 641-8509, Japan

TEL: +81-73-441-0619

E-mail address: nbyamamo@wakayama-med.ac.jp

Supplementary Table S2. Participating principal investigators

**Italy**	• Luca Gianni<br>• Hector Soto Parra<br>• Federico Cappuzzo<br>• Filippo De Marinis
**Spain**	• Susana Cedres<br>• Teresa Morán<br>• Santiago Viteri<br>• Manuel Domine Gomez
**France**	• Jean Charles Soria, David Planchard<br>• Alexis Cortot<br>• Pierre-Jean Souquet<br>• Jeannick Madelaine<br>• Anne Madroszyk<br>• Gerard Zalcman
**USA**	• Howard West<br>• Afshin Dowlati<br>• Dennie Jones<br>• Jonathan Dowell<br>• John J. Nemunaitis, Reva Schneider<br>• Hamid Mirshahidi<br>• Jeremy Cetnar
**Japan**	• Hidetoshi Hayashi<br>• Nobuyuki Yamamoto<br>• Kiyotaka Yoh<br>• Hiroshi Sakai<br>• Yasushi Goto<br>• Makoto Nishio<br>• Akira Ono

Article title: A randomized, phase 2 study of deoxyuridine triphosphatase inhibitor, TAS-114, in combination with S-1 versus S-1 alone in patients with advanced non-small-cell lung cancer

Journal name: Investigational New Drugs

Author names: Nobuyuki Yamamoto, Hidetoshi Hayashi, David Planchard, Teresa Morán, Vanesa Gregorc, Jonathan Dowell, Hiroshi Sakai, Kiyotaka Yoh, Makoto Nishio, Alexis B. Cortot, Karim A. Benhadji, Nital Soni, Jinhong Huang, Lukas Makris, Susana Cedres

Corresponding author: Dr. Nobuyuki Yamamoto, Third Department of Internal Medicine, Wakayama Medical University Hospital, 811-1 Kimiidera, Wakayama, Wakayama Prefecture 641-8509, Japan

TEL: +81-73-441-0619

E-mail address: nbyamamo@wakayama-med.ac.jp

Supplementary Text S1

Eligibility criteria

Inclusion criteria

1. Provision of written informed consent consistent with International Council for Harmonisation-Good Clinical Practice guidelines and respective local law.

2. Age ≥18 years (≥20 years in Japan).

3. Histologically diagnosed or cytologically proven advanced or metastatic non-small cell lung cancer patients, either Stage IIIB/Stage IV disease (according to Version 7 of the International Association for the Study of Lung Cancer Staging Manual in Thoracic Oncology), or recurrent disease following radiation therapy or surgical resection.

4. Patients who had received at least two prior therapies for advanced or metastatic disease condition, including platinum doublet and pemetrexed, docetaxel, or immunotherapy, and were refractory to or unable to tolerate their last prior therapy. For patients with known epidermal growth factor receptor activating mutations or anaplastic lymphoma kinase translocations, and/or ROS proto-oncogene 1 rearrangements, appropriate targeted treatment should have been used. The following histological tumor types could also be included: adenocarcinoma with bronchioloalveolar differentiation and large cell carcinoma.

5. The tumor is locally advanced or metastatic and not suitable for surgery and radiotherapy is not indicated.

6. Measurable disease as defined by Response Evaluation Criteria in Solid Tumors criteria (Version 1.1, 2009).

7. Eastern Cooperative Oncology Group performance status 0 or 1.

8. Predicted life expectancy of at least 3 months.

9. Able to take medications orally.

10. Adequate organ function as defined by the following: adequate bone marrow function (absolute neutrophil count ≥1500/mm^3, hemoglobin ≥10.0 g/dL, platelets ≥100,000/mm^3); adequate liver function (total bilirubin ≤1.5 × upper limit of normal [ULN], aspartate aminotransferase/alanine aminotransferase ≤3 × ULN [in patients with existent liver metastases, ≤5 × ULN]); and adequate renal function (calculated creatinine clearance ≥50 mL/min [Cockcroft-Gault]).

11. Women of childbearing potential must have a negative pregnancy test (urine or serum) within 7 days prior to starting the study drug. Both males and females must agree to use effective birth control during the study (prior to the first dose and for 6 months after the last dose) if conception is possible during this interval. Female patients are considered to not be of childbearing potential if they have a history of hysterectomy, or are post-menopausal defined as no menses for 12 months without an alternative medical cause.

12. Willing and able to comply with required scheduled visits and study procedures.

Exclusion criteria

1. Treatment with any of the following within the specified time frame prior to the study drug administration: major surgery within prior 4 weeks and minor surgery within 7 days; radiotherapy for an extended field within 4 weeks prior or limited field within 2 weeks prior; any anticancer therapy or investigational agent within 3 weeks prior.

2. A serious illness or medical condition including but not limited to the following: patients with brain or subdural metastases are not eligible unless they have completed local therapy and have discontinued the use of therapeutic corticosteroids and are stable for at least 1 month

before study drug administration; known leptomeningeal metastatic disease; known acute or chronic active systemic infection; any cardiac disease, such as myocardial infarction, unstable angina, symptomatic congestive heart failure New York Heart Association class III or IV within the last 6 months. If >6 months, cardiac function must be within normal limits and the patient must be free of cardiac-related symptoms; chronic nausea, vomiting, or diarrhea considered to be clinically significant by investigator's discretion; current or past severe lung disease (e.g., interstitial pneumonia, pulmonary fibrosis, or severe emphysema); pleural, peritoneal, or pericardial effusion which will require surgical intervention in the near term; any history or presence of poorly controlled gastrointestinal disorders that could affect the absorption of the trial drug (e.g., Crohn's disease, ulcerative colitis); known human immunodeficiency virus or acquired immune deficiency syndrome-related illness, or chronic or acute hepatitis B or C; any other clinically significant acute or chronic medical or psychiatric condition or any laboratory abnormality that may increase the risk associated with study drug administration, or may interfere with the interpretation of study results; poorly controlled (despite medication) or severe diabetes mellitus; continuous systemic steroid administration (oral or intravenous); other concurrent active cancer (synchronous double cancer or heterochronous double cancer with a disease-free interval of 3 years or shorter, excluding lesions consistent with intraepithelial cancer, i.e., carcinoma *in situ*, or intramucosal cancer) that is assessed as cured by local treatment.

3. Concomitant treatment with the following drugs that may interact with S-1: sorivudine, brivudine, uracil, eniluracil, folinate/folinic acid (enhance S-1 activity); cimetidine, dipyridamole, and nitroimidazoles, including metronidazole and misonidazole (may enhance S-1 activity); methotrexate (may enhance S-1 activity); clozapine (may increase risk and

severity of hematologic toxicity with S-1); allopurinol (may diminish S-1 activity); phenytoin (S-1 may enhance phenytoin activity); flucytosine, a fluorinated pyrimidine antifungal agent (may enhance S-1 activity); and coumarin-derivative anticoagulant (S-1 may enhance activity of coumarin-derivative anticoagulant).

4. Known hypersensitivity to S-1 or its metabolites (e.g., 5-fluorouracil [5-FU]).

5. Patients with rare hereditary problems of galactose intolerance, Lapp lactase deficiency, or glucose/galactose malabasorption since S-1 contains lactose (only Western countries).

6. Previous use of TAS-114, S-1, and 5-FU drugs.

7. Pregnant or lactating women or possibly pregnant women, or men or women wishing to have children during the study period.

8. Judgment of the investigator that the patient is inappropriate for study participation.

Article title: A randomized, phase 2 study of deoxyuridine triphosphatase inhibitor, TAS-114, in combination with S-1 versus S-1 alone in patients with advanced non-small-cell lung cancer

Journal name: Investigational New Drugs

Author names: Nobuyuki Yamamoto, Hidetoshi Hayashi, David Planchard, Teresa Morán, Vanesa Gregorc, Jonathan Dowell, Hiroshi Sakai, Kiyotaka Yoh, Makoto Nishio, Alexis B. Cortot, Karim A. Benhadji, Nital Soni, Jinhong Huang, Lukas Makris, Susana Cedres

Corresponding author: Dr. Nobuyuki Yamamoto, Third Department of Internal Medicine, Wakayama Medical University Hospital, 811-1 Kimiidera, Wakayama, Wakayama Prefecture 641-8509, Japan

TEL: +81-73-441-0619

E-mail address: nbyamamo@wakayama-med.ac.jp

Supplementary Text S2

Exploratory analysis results: Progression-free survival (PFS) for patients with Grade ≥1 anemia or Grade ≥1 rash in the TAS-114/S-1 group

Among patients with rash (including those with rash, rash maculo-papular, rash erythematous, and rash papular), the median PFS was 5.16 months in the Grade ≥1 group and 2.66 months in Grade 0 group (hazard ratio [HR] 0.41, 95% confidence interval [CI] 0.20–0.83; *P*=0.0107). Among patients with anemia, the median PFS was 4.21 months in the Grade ≥1 group and 2.66 months in the Grade 0 group (HR 0.53, 95% CI 0.27–1.04; *P*=0.0587).

Article title: A randomized, phase 2 study of deoxyuridine triphosphatase inhibitor, TAS-114, in combination with S-1 versus S-1 alone in patients with advanced non-small-cell lung cancer

Journal name: Investigational New Drugs

Author names: Nobuyuki Yamamoto, Hidetoshi Hayashi, David Planchard, Teresa Morán, Vanesa Gregorc, Jonathan Dowell, Hiroshi Sakai, Kiyotaka Yoh, Makoto Nishio, Alexis B. Cortot, Karim A. Benhadji, Nital Soni, Jinhong Huang, Lukas Makris, Susana Cedres

Corresponding author: Dr. Nobuyuki Yamamoto, Third Department of Internal Medicine, Wakayama Medical University Hospital, 811-1 Kimiidera, Wakayama, Wakayama Prefecture 641-8509, Japan

TEL: +81-73-441-0619

E-mail address: nbyamamo@wakayama-med.ac.jp
